# Neural Circuitry of Emotional and Cognitive Conflict Revealed through Facial Expressions

**DOI:** 10.1371/journal.pone.0017635

**Published:** 2011-03-09

**Authors:** Kimberly S. Chiew, Todd S. Braver

**Affiliations:** Department of Psychology, Washington University in St. Louis, St. Louis, Missouri, United States of America; University of Leuven, Belgium

## Abstract

**Background:**

Neural systems underlying conflict processing have been well studied in the cognitive realm, but the extent to which these overlap with those underlying emotional conflict processing remains unclear. A novel adaptation of the AX Continuous Performance Task (AX-CPT), a stimulus-response incompatibility paradigm, was examined that permits close comparison of emotional and cognitive conflict conditions, through the use of affectively-valenced facial expressions as the response modality.

**Methodology/Principal Findings:**

Brain activity was monitored with functional magnetic resonance imaging (fMRI) during performance of the emotional AX-CPT. Emotional conflict was manipulated on a trial-by-trial basis, by requiring contextually pre-cued facial expressions to emotional probe stimuli (IAPS images) that were either affectively compatible (low-conflict) or incompatible (high-conflict). The emotion condition was contrasted against a matched cognitive condition that was identical in all respects, except that probe stimuli were emotionally neutral. Components of the brain cognitive control network, including dorsal anterior cingulate cortex (ACC) and lateral prefrontal cortex (PFC), showed conflict-related activation increases in both conditions, but with higher activity during emotion conditions. In contrast, emotion conflict effects were not found in regions associated with affective processing, such as rostral ACC.

**Conclusions/Significance:**

These activation patterns provide evidence for a domain-general neural system that is active for both emotional and cognitive conflict processing. In line with previous behavioural evidence, greatest activity in these brain regions occurred when both emotional and cognitive influences additively combined to produce increased interference.

## Introduction

‘Cognitive control’ refers to the coordination and direction of lower-level cognitive processes critical to complex, goal-directed behaviour. These processes, including attentional selection, conflict resolution, and the online maintenance of goal-relevant information (and inhibition of goal-irrelevant information), may underlie higher many cognitive functions, permitting the flexibility and sophistication of human thought and behaviour across a wide variety of task situations. While cognition has traditionally been conceptualized as separate from affect, it has been increasingly recognized that affective significance is a major factor in goal-directed behaviour, both in establishing goals and in shaping how information is processed during goal pursuit. Emotionally salient stimuli in the environment may be prioritized for processing over non-emotional stimuli [1], [2], but it remains unclear whether qualitatively distinct neural circuitry is engaged for the processing of affectively-valenced stimulus dimensions. The present study examines the neural systems engaged in the detection and management of conflict, a canonical control function, when the information being processed (i.e., the source of conflict) is emotional versus non-emotional in nature.

*Conflict* can be defined mechanistically in terms of cross-talk caused by the simultaneous concurrent processing of goal-relevant and goal-irrelevant information competing for common resources [3]. The Stroop task [4] is a classic conflict task: participants must name the colour of presented words while ignoring the word's meaning. In some trials, goal-irrelevant information is congruent with goal-relevant information (e.g., the word ‘RED’ printed in red ink); in other trials, the goal-relevant and irrelevant information are incongruent (e.g., ‘RED’ printed in green ink), leading to conflict. Using tasks such as the Stroop (as well as related incompatibility paradigms such as Simon, flanker and others, e.g., [5]), conflict has been extensively studied in the cognitive realm. Functional neuroimaging methods have been used to identify a number of frontal and parietal brain regions canonically associated with cognitive control in this and other tasks [6], [7], [8], [9], with the ACC in particular being associated with conflict processing functions [10], [11], [12], [13].

Evidence from early neuroimaging studies examining conflict elicited by emotional versus non-emotional distracters resulted in an influential hypothesis postulating that emotional and cognitive conflict detection are mediated by distinct rostral and dorsal subdivisions of the ACC, respectively (Bush et al., 2000). However, subsequent investigations of emotional and cognitive conflict processing have yielded mixed evidence regarding the domain-specificity of their underlying neural systems. Most studies focusing on emotion conflict using Stroop-like variants tend to find activation in dorsal rather than ventral ACC, as well as other areas associated with cognitive control, such as the lateral PFC [14], [15]. In a recent study comparing activity in closely matched emotion and non-emotional variants of a face-word Stroop paradigm, Egner and colleagues [16] again reported that conflict detection was associated with dorsal ACC in both conditions; activation was observed in the rostral ACC (and amygdala) only during conditions examining emotional conflict resolution (i.e., modulation based on previous trial conflict). Ochsner and colleagues [17] compared an emotional versus non-emotional flanker task, and also found a number of areas commonly engaged by conflict in both tasks, including the dorsal ACC. However, consistent with the cognitive/emotion division hypothesis, they also observed that affective conflict selectively engaged the rostral medial PFC, with brain-behavior correlations observed in rostral ACC. Likewise, another recent study [18] reported distinct patterns of conflict-related neural activity in conditions involving emotional stimulus-response (S-R) incompatibility (emotion expression interference; elicited via making facial expressions incongruent with those of presented faces) with cognitive S-R incompatibility (elicited via the Simon task).

A major challenge in this research area has been to utilize appropriate paradigms that enable valid and closely matched comparisons of emotional and cognitive forms of conflict. The hypothesis that emotional versus cognitive conflict may depend on distinct subdivisions of the ACC was based on evidence from emotional adaptations of the Stroop task, which examine interference from emotional distracters (e.g., performance of the colour-naming task for emotional relative to non-emotional words; [19]). However, it has been asserted that interference in the colour-naming emotional Stroop task may occur because of lower-level lexical effects [20] or general attention capture [21] rather than the direct conflict effects present in the traditional Stroop. To improve upon this design, face-word Stroop variants have been utilized, in which positive and negatively valenced words (e.g., ‘HAPPY’ or ‘FEAR’) are superimposed on compatible or compatible facial expressions [15], [16], [22], [23]. This design improves on the colour-naming emotional Stroop in that the responses require affective classification and the task-relevant and irrelevant information are semantically related, leading to affective incompatibility effects more closely related to the direct conflict present in the traditional cognitive Stroop. However, all of these tasks involve an incompatibility between a task-relevant stimulus and a task-irrelevant stimulus (thus, stimulus-stimulus [S-S] incompatibility). In contrast, studies of cognitive conflict have explored both S-S and S-R incompatibilities [5]. The emotion expression interference paradigm developed by Lee and colleagues [18] is a first step in exploring S-R incompatibility in the context of emotional conflict: this paradigm examines interference when participants make emotional facial expressions as a behavioral response, capitalizing on their role as an index of emotional experience and expression [24]. However, the Lee et al paradigm requires participants to make an expression in response to a presented face. As such, it leaves open the possibility that interference effects in the task may be caused by overriding imitation tendencies instead of being due to conflicting emotional influences, per se. In view of these considerations, our goal was to examine emotional conflict with a paradigm that similarly capitalized on emotional facial expressions to index stimulus-response incompatibility, but that improved upon this paradigm by avoiding possible imitative influences.

Accordingly, we developed a new paradigm to examine emotional conflict via S-R incompatibility using emotional facial expressions to emotional, but non-face stimuli [25]. This task was adapted from the *AX Continuous Performance Task* (AX-CPT), which has been repeatedly established as a robust probe of context processing, cognitive conflict, and cognitive control [13], [26], [27], [28]. The emotional AX-CPT requires participants to respond to emotionally evocative cue-probe combinations with emotionally congruent or incongruent facial expressions. This task was developed on the rationale that interference elicited by a mismatch between evoked emotion and required facial response may more closely approximate situations of emotional conflict that people experience in ‘real-life’ (e.g., acting pleasant to a rude customer; smiling graciously after a defeat), thus achieving a higher level of ecological validity. In prior work using facial electromyography (EMG) to index expression responses in this task, we demonstrated that behavioural interference can be robustly elicited, and furthermore, that such interference was greater when emotional influences were present relative to when they were absent [25].

In the AX-CPT, conflict and cognitive control are varied on a trial-by-trial basis through the use of contextual pre-cues. Certain cue-probe combinations require a target response (e.g., ‘A’ followed by ‘X’), whereas all other cue-probe combinations require a non-target response. The target (‘AX’) combination occurs with high frequency, which leads to high levels of interference in two low-frequency cue-probe combinations: AY (target cue, non-target probe) and BX (non-target cue, target probe). In AY trials, interference arises from expectancy established by the target cue, while in BX trials interference arises via a dominant target response bias to the probe. In both trial combinations, target-related response biases produce stimulus-response interference because a non-target response is required. In the emotional AX-CPT we developed, text instructions (‘SMILE’ and ‘FROWN’) were used as cues and emotionally evocative pictures (from the International Affective Picture System [IAPS];[29] served as probes; participants were required to smile or frown in response. The target cue-probe-response combination was always emotionally congruent (i.e., smiling to ‘SMILE’+pleasant picture, or frowning to ‘FROWN’+unpleasant picture). BX trials (non-target cue, target probe) involved incompatibility between the probe presented and the required facial response (e.g., smiling to an unpleasant picture); in contrast, interference in AY trials (target cue, non-target probe) was due to incompatibility between the instructions of the cue and the required facial response (e.g., frowning after ‘SMILE’ cue). When contrasting performance in the emotion AX-CPT relative to a tightly matched non-emotional condition (in which probes were emotionally neutral), utilizing EMG measures to quantify the facial expression response, we observed that interference effects were present under both emotional and non-emotional conditions, but were strongest in the emotional AX-CPT, when both emotional and non-emotional sources of incompatibility were present [25]. In this condition, interference was due not only to standard sources of S-R incompatibility, but also because of the automatic, but inappropriate affective response to the target (e.g. being cued to smile to a negative IAPS picture).

This paradigm is unique among present tasks probing emotional conflict, in that it requires integrated processing of *both* cue and probe in order to perform successfully, as opposed to requiring inhibition of the emotional information. Additionally, a major strength of the paradigm is the ability to create a closely matched analog task that permits a direct comparison of emotional vs. non-emotional conflict. Specifically, by changing probe stimuli to be affectively neutral (i.e., arbitrary symbol categories instead of emotionally evocative pictures), but retaining the other aspects of the task structure (including using facial expressions as response modality), sources of S-R incompatibility in the affective dimension are eliminated, while the standard non-affective S-R association effects driving AX-CPT effects remain (i.e., probe-driven biases and cue-driven expectancies). By comparing effects in the two conditions, it is possible to isolate the additive conflict effects specifically associated with S-R incompatibility in the affective dimension.

The present study builds on our previous behavioural work by using event-related fMRI to examine whether brain activity associated with processing emotional vs. non-emotional conflict involves the same general control-related regions or qualitatively different neural circuits. Such a comparison may help to clarify further some of the outstanding contradictions present in previous emotion conflict research. On the basis of previous neuroimaging evidence, we hypothesized that both emotional and non-emotional versions of this task would engage common control-related regions including the dorsal ACC and lateral PFC. Further, based on our previous behavioural evidence, we predicted that conflict-related interference would be greater in the emotional task than in the non-emotional task, and that this would be reflected in increased levels of elicited activity within these control-related brain regions. Finally, we tested whether the emotional task was associated with the activation of potentially affectively-specialized regions, such as the rostral ACC/ventromedial PFC and amygdala, that might be selectively recruited to detect emotional conflict.

## Methods

### Ethics Statement

Ethics approval to conduct this study was granted by the Institutional Review Board of Washington University. Each participant provided written, informed consent prior to participation, in accordance with the human subjects guidelines established by Washington University.

### Participants

Twenty-four healthy young adults (8 males, 16 females; mean age = 25.5 years, SD = 5.63) were scanned using fMRI while participating in the task. All fMRI participants were right-handed, native English speakers, and screened to ensure no neurological or psychiatric disorders, psychotropic medications, or other factors were present that contraindicated fMRI.

### Task Procedure

Participants performed an emotional (Emotion condition) and non-emotional (Neutral condition) variant of the AX-CPT. The AX-CPT paradigm follows a cue-probe trial structure, in which cue stimuli set a context that is needed for appropriate response selection to the subsequent probe. The Emotion and Neutral conditions were identical in all respects except for the category of stimuli used as probes. Cue stimuli in the task were the words ‘SMILE’ and ‘FROWN’. For probes, the Emotion condition used IAPS pictures as probes and the Neutral condition used alphanumeric symbols (i.e., letters served as target probes, and digits served as nontarget probes). New pictures/symbols were used as probes on each trial, except for a pre-specified neutral picture/punctuation mark on no-go trials (described below). Across participants the particular cue-probe combination that comprised the “AX” target trial type was counter-balanced. Thus, for approximately half of the participants (11/24) the AX target was ‘SMILE’/positive picture (“SMILE”/letter in Neutral) requiring a smile response (facial expression) and the other half (13/24) the AX target was ‘FROWN/negative picture (“FROWN”/letter in Neutral) requiring a frown response. However, on nontarget trials (AY, BX, BY), the opposite facial expression was required. All other details of the task paradigms described below were identical for the Emotion and Neutral conditions, and for both participant groups.

Trials were presented in pseudorandom sequence, with target (AX) trials occurring at a 7∶1 frequency compared to all non-target task trials, leading to a total of 84 AX trials, 12 AY trials (target cue, non-target probe), 12 BX trials (non-target cue, target probe), 12 BY trials (non-target cue, non-target probe). Although the absolute numbers of high conflict (BX and AY) trials is somewhat low, our prior results suggest that this number was sufficient to robustly detect significant interference effects. In addition to primary task trials, no-go trials were also included to ensure that participants responded on the basis of the cue-probe combination and not solely and prematurely to the cue. No-go trials were indicated by a pre-specified neutral picture in Emotion (punctuation mark in Neutral), to which no response was to be made (24 no-go trials total; occurring both after target and non-target cues).

**Figure 1:**
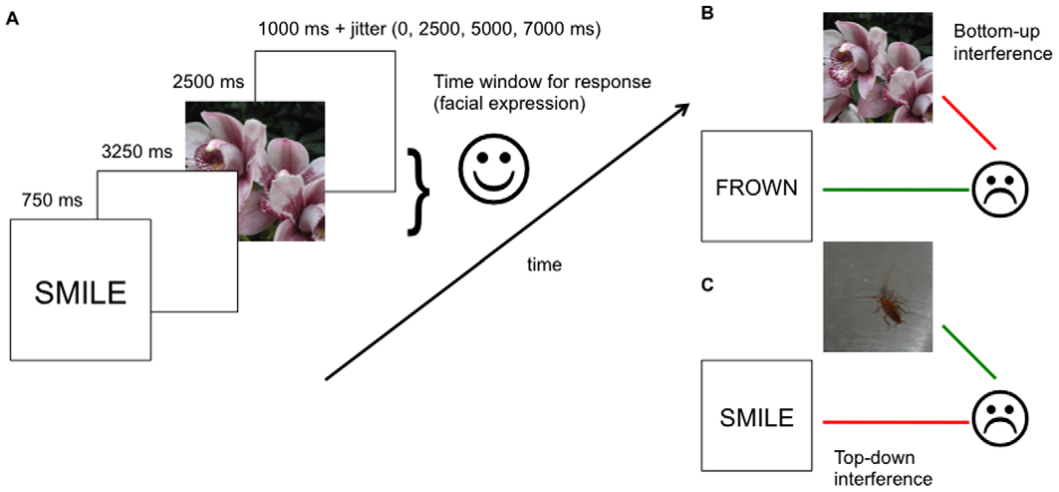
Trial structure with timing. (A) Example of the target (AX) cue-probe-response for the smile-AX condition of the Emotion AX-CPT; (B) Example of BX (non-target cue, target probe) and (C) AY (target cue, non-target probe) conflict trials for smile-AX condition of the task.

Participants performed four scanning runs each of the Emotion and Neutral conditions of the AX-CPT (eight runs in total). Within each run, task blocks (three per run; 135 seconds each) alternated with short fixation blocks (four per run; 30 seconds each). Each scanning run began with 10 seconds of rest (later discarded) to allow the scanner to reach steady state; total run duration was ∼9 minutes. Each of the three task blocks within a scanning run consisted of 12 trials; thus participants performed eight runs of 36 AX-CPT trials each for 288 trials in total (144 Emotion, 144 Neutral). AX-CPT trials consisted of cue-probe pairs shown in sequence. Trial structure (Figure 1) was as follows: cue (750 ms), inter-stimulus-interval (ISI; 3250 ms), probe (2500 ms), and minimum inter-trial-interval (ITI) of 1000 ms (for a minimum total trial length of 7.5 seconds). ITIs included additional jittering to facilitate event-related response estimation, in increments of 2500 ms (no jitter, 2500 ms, 5000 ms, or 7500 ms). 72 trials were presented at each of the four ITI lengths.

### fMRI Data Collection

Structural and functional imaging data was collected on a 3T Siemens TIM Trio whole-body scanner at Mallinckrodt Institute of Radiology at Washington University School of Medicine. High-resolution anatomical images were acquired for each participant using a sagittal T1-weighted MP-RAGE sequence (TE = 3.16 ms, TR = 2400 ms, flip angle = 8° 176 slices, 1×1×1 mm voxels). Anatomical images were aligned with each individual's functional images. To facilitate registration of the T1 and functional scans, a T2-weighted image was also acquired in the same space as the functional scans [TE = 96 ms, TR = 5000 ms, 189×256 acquisition matrix, 48 slices, 1.02×1×3 mm voxels]. The functional images were collected in eight 210TR (∼9 minutes) runs using an asymmetric spin-echo echo-planar sequence sensitive to blood oxygenation level-dependent (BOLD) contrast (T2*) [TE = 27 ms, TR = 2500 ms, flip angle = 90°, FOV = 256 mm, skip = 0 mm, 36 slices, 4×4×4 mm voxels].

Stimuli were presented using E-Prime (Psychology Software Tools, Pittsburgh, PA) on a Dell PC. As described in the Task Procedure section, participants responded to each trial using emotional facial expressions. A custom-built mirror apparatus positioned over the head coil served both to reflect the projected image of the task screen towards the participant and to reflect the view of the participant's face such that it could be recorded using a videocamera positioned at the head end of the bore. Video recording served to ensure participant compliance in the task and was visually inspected to verify that such compliance was occurring. However, due to technical difficulties and poor video quality, this video was not quantitatively evaluated for measures of behavioural performance. A fiber-optic button box interfaced with E-Prime facilitated communication with the participant.

### fMRI Data Analysis

The fMRI data were analyzed with in-house software. Data analysis was conducted with a general linear model (GLM), including nuisance regressors for linear trends within runs and baseline shifts between runs. Additionally, the GLM contained task-related regressors for block and event-related activity. Block-related activity related to each task condition (Emotion and Neutral) were modeled as boxcar functions, but because examining sustained activity did not permit the examination of conflict effects in the data, these functions were also treated as regressors of no interest. Our experimental design follows the specifications of Visscher et al. [30] in permitting the dissociation of block and event-related fMRI activity (using alternating blocks of task and rest, as well as jittered trials within each task block); using event-related regressors that are estimated (using delta or FIR functions) rather than assumed via a model of the hemodynamic response function. With this estimation approach, multicollinearity between the sustained and event-related regressors has been shown not to be a major concern.

The primary task-related analysis focused on event-related activity as a function of trial type and task condition. Event-related estimates were created for each trial type within task conditions (AX, AY, BX, BY, no-go within Emotion and Neutral task versions). Given the complex trial structure, event-related effects were analyzed without reference to a fixed hemodynamic response function, using a delta-function estimation approach. Thus, within a 25-second response epoch following trial onset, independent values were estimated for each of 10 timepoints (corresponding to the 10 TR frames). The estimates from the individual subject GLMs were analyzed using appropriately designed analyses of variance (ANOVAs) that treated participants as a random factor.

#### Regions of interest identification

We examined event-related brain activity in analyses within *a priori* defined regions of interest (ROIs). Analyses were conducted within two ‘networks’ of interest (selected not on the basis of functional connectivity but as coherent sets of regions observed in prior literature to be functionally related to cognitive control and reward processing). The first analysis examined activity within regions associated with cognitive control and working memory (established using meta-analyses; primarily including dorsal medial and lateral prefrontal and parietal regions [8], [9]. The ROI mask for the cognitive control network (CCN) was created by using anatomical coordinates identified by the aforementioned meta-analyses as seed points with 10 mm radius spheres drawn around them. The second analysis examined activity within anatomical regions associated with emotion and reward processing (hereafter EMO network) including the amygdala, portions of the basal ganglia (putamen, caudate, substantia nigra and nucleus accumbens), anterior insula, medial orbitofrontal cortex, and ventromedial prefrontal cortex, with regions drawn according to anatomical criteria identified using the Talaraich atlas [31], and previous studies [32], [33], [34], [35], [36], [37], [38], [39], [40]. A separate region of interest included the rostral ACC, defined anatomically, which was near to, but not overlapping the ventromedial PFC ROI [41]. For coordinates for ROIs in both networks, please refer to Table S1 and Table S2. The exact masks for both networks are available from the authors by request.

Significant activity within each network mask was corrected for multiple comparisons using a cluster size criterion based on Monte Carlo simulations [42], [43], via the AlphaSim software within AFNI [44]. To assure a multiple comparisons corrected *p*<.05 criteria, significant regions were identified based on a per-voxel minimum *z*>2.32 and minimum cluster size of 37 voxels within the CCN mask (or 30 voxels within the EMO mask).

Within each mask, we were interested in identifying regions demonstrating general sensitivity to conflict (e.g., across both the emotional and non-emotional tasks) and then examining whether brain activity within these conflict-associated regions differed as a function of emotional task content. Thus, the first stage analysis consisted of the following voxelwise contrast: high conflict trials (AY + BX collapsed, averaged across timepoints 4–7) > low conflict trials (AX + BY collapsed, averaged across timepoints 4–7). This analysis further collapsed across the Emotion and Neutral conditions, in order to enable unbiased identification of regions. Timepoints 4–7 were selected to capture probe-related activity, which is necessary for the elicitation of conflict in the AX-CPT paradigm.

In the second stage of analysis, we conducted ROI-based ANOVAs on significant regions identified as sensitive to conflict in the first-stage analysis. Two different kinds of region-wise analyses were carried out. In the first ANOVA, we examined which, if any, of these conflict-defined regions showed independent differences in brain activity as a function of task condition, i.e., Emotion vs. Neutral, and time (the ANOVA included all 10 timepoints). This analysis enabled a direct test of whether conflict-related regions showed increased responsivity under emotion conditions. In the second ANOVA, we only included the high-conflict trials AY and BX, to examine whether task condition effects were still exhibited selectively during conflict. Additionally, by including trial-type as a factor, we tested whether condition effects differed by the type of conflict elicited (AY = cue-based; BX = probe-based; again, timepoint was also included as a factor in the ANOVA).

In addition to analyses within these networks of interest, we conducted more focused analyses within the rostral ACC and amygdala ROIs, as these regions have been specifically implicated in emotional conflict processing [16], [45]. The amygdala was part of the general EMO mask, but the additional analyses focused exclusively on amygdala and rACC regions, and as such utilized a more liberal corrected threshold specific to the size of each ROI (i.e., small-volume correction). Thus, for these analyses a reduced cluster-size criterion of 12 voxels for rostral ACC and 9 voxels for amygdala was employed (again with voxelwise minimum *z*>2.32). In addition to the analyses described above, we also conducted a focused test with the rACC and amygdala ROIs to examine whether these regions show a selective response to conflict only under Emotion conditions. As such, a voxelwise contrast of high conflict (AY + BX, timepoints 4–7) > low conflict (AX + BY, timepoints 4–7) was conducted, but only using trials from the Emotion condition.

## Results

### Behavioural Performance

As described in Methods, participants performed the emotional AX-CPT with voluntary emotional facial expressions as the response modality. Facial expressions were monitored in the scanner using video recording and video footage was inspected following each participant to ensure compliance with the task, but poor video quality and technical difficulties rendered this video unusable for the purposes of evaluating behavioural performance.

Previously published data from our laboratory [25] investigating the Emotion and Neutral versions of the AX-CPT used here found no significant main effects of task condition on performance (indexed by error rates and response onsets), suggesting that the overall difficulty of emotional and non-emotional versions of the task may be comparable. Additionally, that study indexed performance using facial electromyography (EMG), which enables a much more fine-grained behavioural analysis than video coding would have permitted in the present study. We discuss issues with the present study's behavioural data and present behaviour from our previous EMG study in Text S1 and Figure S1. We compared areas defined by task conflict (high > low conflict) within the CCN and EMO networks with and without discernable errors, and found relatively few differences. These results are shown in Table S3.

### Imaging Results: ROI Analyses

As described in the Methods section, event-related brain activity was examined within two ‘networks’ of interest: the cognitive control network (CCN) and the emotion/reward processing network (EMO). We also analyzed brain activity within more focused ROIs of the rostral ACC and bilateral amygdala.

#### Conflict-defined regions within CCN and EMO ROIs

**Figure 2:**
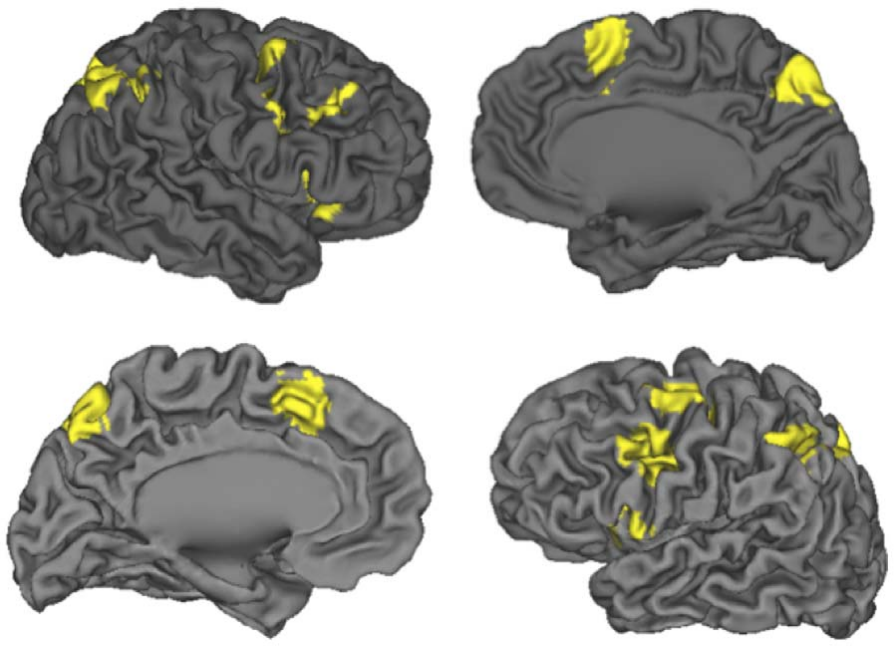
Cortical areas sensitive to the High > Low conflict contrast. These areas fall within the CCN and REW masks and were identified as showing significant (AY + BX) > (AX + BY) activation, collapsed across Emotion and Neutral conditions.

Within each ROI, we identified regions showing conflict-related increases in activity through the high-conflict > low-conflict contrast, collapsing across Emotion and Neutral conditions to provide an unbiased test. Fourteen regions within the CCN, as well as five regions within the EMO network, were identified as showing conflict responses. These conflict-defined regions are summarized in Table 1, with cortical regions shown in Figure 2. As expected, conflict-related regions within the CCN included the dorsal ACC and bilateral PFC, along with additional activation in the inferior parietal lobule, precuneus, thalamus, and cerebellum. The EMO regions showing sensitivity to conflict included bilateral dopaminergic midbrain, bilateral anterior insula, and left putamen. However, in this contrast, conflict-related activation was not observed in ventromedial PFC or amygdala.

**Table 1:** Activity in areas defined by task conflict (high>low conflict) within anatomically defined ROIs.

Coordinates	Cluster Size (mm^3^)	ROI	Z	BA	Area	Sig. Condition*Time effect in High vs. Low Conflict Contrast	Sig. Condition*Time effect in AY vs. BX Trial Contrast	Sig. Condition*Time effect in AY vs. BX Trial Contrast
0, 11, 48	7722	CCN	3.63	32	Dorsal ACC	*	*	*
41, 28, 35	1512	CCN	2.88	9	R DLPFC	*	*	*
−44, 8, 33	5589	CCN	3.48	9	L IFJ	*	*	*
45, 5, 32	2673	CCN	2.93	9	R IFJ	*	*	*
−49, 13, 3	2457	CCN	3.20	47	L IFG			*
28, 0, 54	7155	CCN	4.15	8	R superior frontal (FEF)	*	*	
−28, −1, 55	7938	CCN	3.83	8	L superior frontal (FEF)			
18, −60, 43	23031	CCN	4.83	7	R precuneus			*
−37, −52, 40	6534	CCN	4.24	40	L IPL	*		*
10, −12, 4	3915	CCN	3.88	----	R thalamus			*
−9, −11, 6	1215	CCN	2.89	----	L thalamus			*
−31, −67, −45	2052	CCN	3.08	----	L cerebellum			
32, −60, −44	1215	CCN	3.07	----	R cerebellum			
33, −62, −26	999	CCN	4.27	----	R cerebellum	*	*	*
39, 20, 0	3996	EMO	4.83	47/13	R anterior insula	*		
−36, 17, 0	3051	EMO	4.02	47/13	L anterior insula	*	*	
−16, 5, −2	1161	EMO	3.36	----	L putamen		*	*
8, −17, −10	1026	EMO	3.12	----	R DA midbrain			
−6, −18, −10	1242	EMO	3.35	----	L DA midbrain			
−16, −1, −11	324	EMO	2.84	----	L amygdala	*	*	

1 Significant effects of interest within these areas (condition*time interactions within high vs. low conflict contrast and AY vs. BX trials contrast; trial*time interactions within AY vs. BX trials contrast) are marked by asterisks in their respective columns.

2 Abbreviations: ROI = region of interest; CCN = cognitive control network; EMO = emotion/reward network; BA = Brodmann area; IFG = inferior frontal gyrus; IFJ = inferior frontal junction; DLPFC = dorsolateral prefrontal cortex; ACC = anterior cingulate cortex; IPL = inferior parietal lobule; DA = dopaminergic.; FEF = frontal eye fields.

#### Condition-related effects within the high versus low conflict contrast

**Figure 3:**
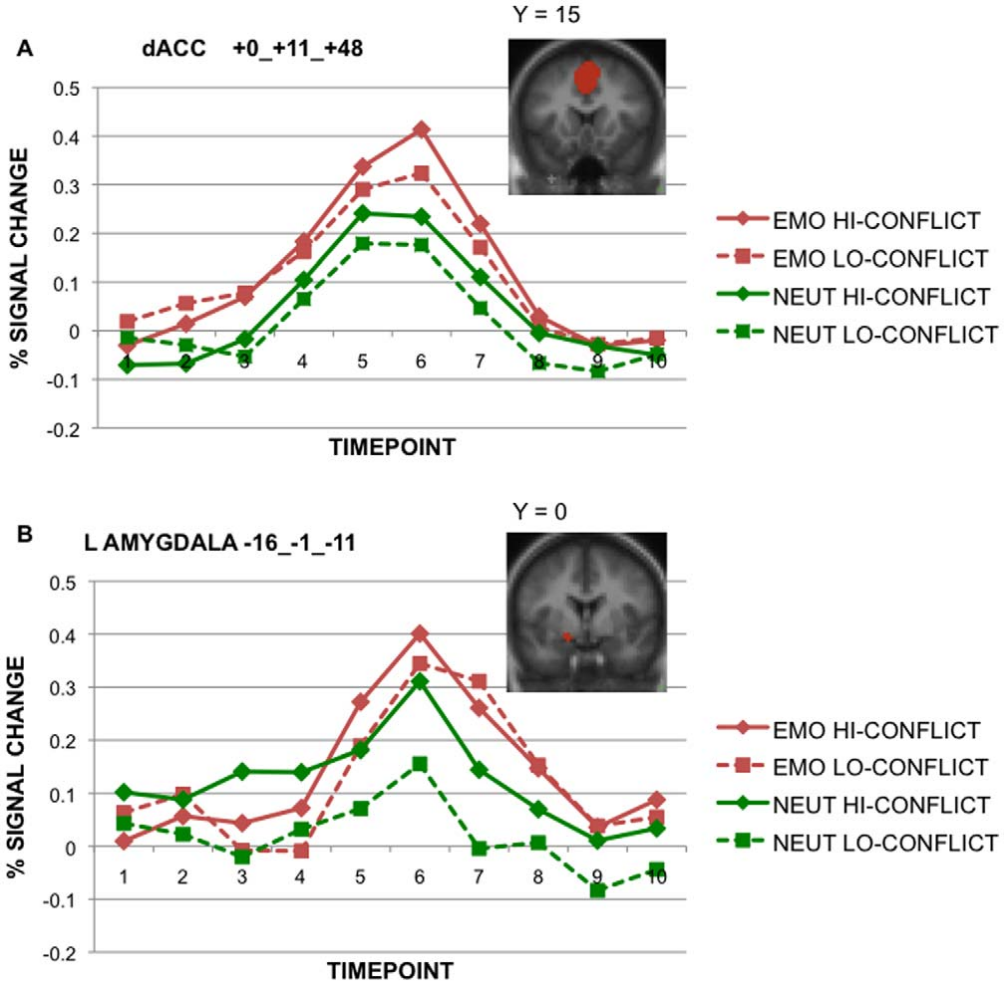
Timecourses illustrating High > Low Conflict and Emotion > Neutral effects. Representative regions demonstrating both a high > low conflict and Emotion > Neutral pattern (due to a condition*time interaction), but no conflict* condition interaction: (A) dorsal ACC; (B) left amygdala.

In the next stage of analysis, each of these conflict-defined ROIs was subjected to an ANOVA that tested for effects of condition type, using timepoint as an additional factor to define event-related effects (i.e., in terms of a condition × time interaction). Nine ROIs showed such condition × time effects – these areas are marked in a column in Table 1. The areas showing sensitivity to both conflict and emotional task content included, most prominently, the dorsal ACC, right dorsolateral PFC, and bilateral posterior PFC, near the inferior frontal junction. The examination of timecourses in these nine regions revealed that, in all of them, the condition × time interaction was due to Emotion > Neutral activation, especially in the middle timepoints where activity peaked (approximately timepoints 4–7). The timecourse of the effect within the dorsal ACC is shown in Figure 3, as a representative illustration of this pattern. In this and the other regions, the effects of condition did not interact with conflict, but instead were present as an additive increase in activation. In only one region, the right dopaminergic (DA) midbrain, was there evidence of a condition*conflict interaction (at trend-level, *p* = .057). However, this interaction was due to increased activity in both the high and low conflict trials of the Emotion condition (i.e., with a reduced conflict-related increase), compared to the Neutral condition.

#### Emotion and trial-type effects under high conflict

Because the ANOVA described above showed Emotion effects that did not interact with conflict, we conducted a follow-up ANOVA to address two additional questions: 1) Was the Emotion-related increase in activation present even when only considering high-conflict trials (i.e., AY and BX)? 2) Were there any differential effects of Emotion related to the type of conflict experienced, i.e., cue-based (AY trials) versus probe-based (BX trials)? To address these questions, the second ANOVA included only the high-conflict trial types (AY, BX) and excluded the low-conflict trials (AX,BY), to examine potential effects of condition (Emotion, Neutral) and high-conflict trial-type (AY,BX) as primary factors of interest (additional factors again included timepoint, and target expression).

The primary pattern observed in the first ANOVA, a condition × time interaction, was replicated in the second ANOVA. Eight regions showed this effect, denoted in Table 1; again, these included dorsal ACC, right dorsolateral PFC, and bilateral PFC regions. Importantly, the same Emotion > Neutral pattern was observed in these regions, confirming that high emotion-conflict trials increased activation of the cognitive control system relative to non-emotion conflict conditions.

**Figure 4:**
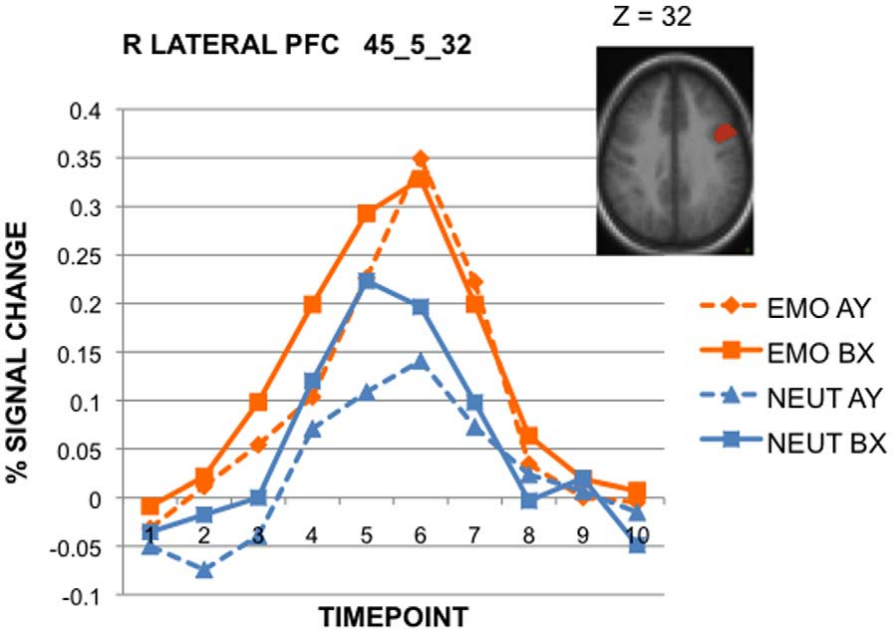
Timecourse illustrating BX > AY trial-type effect. Representative region in right lateral PFC exhibiting BX > AY activity early, and comparable levels of activity in both trial types later in the timecourse.

A second pattern that was observed in the ANOVA was a trial-type × time interaction, which was significant in 11 ROIs. In all of these regions, the pattern was due to increased activation on BX trials relative to AY, during the early part of the trial (timepoints 2–5), but then comparable activation later in the trial (timepoints 6–10). Figure 4 demonstrates this timecourse pattern in an example region, the right lateral PFC. Although a BX > AY pattern is consistent with conflict being increased under probe-based conditions, the early, rather than late timecourse of the effect suggests that the trial-type effect might be anticipatory or expectancy-related. Note that the expectancy for high conflict is significantly greater following a B-cue (probability BX | B-cue∼0.4) than following an A-cue (probability AY | A-cue∼0.1). Thus, differential conflict anticipation or expectancy may account for the trial-type effects, rather than a differential response to experienced conflict during probe processing. Similar conflict expectancy effects have been observed in prior studies of the AX-CPT [46], [47] and other conflict paradigms [48].

Although effects of condition and trial-type were present, the two factors did not appear to interact, as no regions showed evidence of condition × trial-type or condition × trial-type × time interactions. Thus, the BX > AY pattern did not differ significantly between Emotion & Neutral conditions.

#### Focused analysis of rostral ACC and amygdala activity: conflict and condition effects

As done previously within the CCN and EMO masks, we computed contrasts (high > low conflict) within the rostral ACC and amygdala ROIs. However, to test whether these regions were particularly sensitive to emotion conflict per se, we conducted a follow-up ANOVA using a high > low conflict contrast, but restricting to the Emotion condition only. No voxels within the rostral ACC or amygdala survived this contrast, contrary to evidence from previous studies suggesting their sensitivity to emotional conflict.

As a final test to ensure that we did not produce any false negatives, we tested the high > low conflict contrast, using all the data (Emotion and Neutral), but with lowered statistical thresholds, utilizing a small-volume correction for each region individually. Even with these more liberal thresholds, no rostral ACC clusters were observed; however, a small voxel cluster within the left amygdala was identified (see Table 1). Within this conflict-sensitive left amygdala region, there was a significant effect of task condition in the full ANOVA (i.e., involving conflict, condition and timepoint as factors; see Figure 3). This interaction was due to a similar pattern of activity to that observed in several other regions within the CCN and EMO networks (i.e., Emotion > Neutral activity). Similarly, as with these other regions, no condition*conflict interaction was observed. Indeed, if anything, the high > low conflict effect was weaker in the Emotion condition relative to Neutral (Figure 3), consistent with the absence of a significant conflict effect in this region when only the Emotion condition was examined. Additionally, in this left amygdala region, no effects of trial type were observed in the ANOVA contrasting AY and BX trials. Together, these results confirm that the rostral ACC and amygdala did not show any selective emotion conflict effects, and the small left amygdala region that was identified showed a pattern of activation that was very similar to other regions within cognitive control network, i.e., sensitivity to both to the presence of task conflict and to emotional processing, but no preferential response to emotional conflict (e.g., these factors did not interact with one another).

## Discussion

With the present study, we examined neural activity associated with emotional versus non-emotional conflict using a novel paradigm: the emotional AX-CPT. This paradigm capitalized on the use of controlled facial expressions as a response modality to generate S-R incompatibility that was either emotional or non-emotional in nature. The examination of brain activity associated with the processing of these two forms of S-R incompatibility helps clarify the extent to which emotional conflict relies on neural circuitry common to that associated with more traditionally studied forms of cognitive conflict. Specifically, the current findings suggest that both emotional and non-emotional conflict commonly engage a number of brain regions associated with cognitive control, including the dorsal ACC and lateral PFC, as well as certain areas implicated in both emotional processing and cognitive control, such as bilateral anterior insula. Additionally, most of these common regions demonstrated higher activity when processing emotional (versus non-emotional) conflict; in contrast, we observed no conflict-sensitive regions where the non-emotional task elicited greater activity than the emotional task.

Our findings are in line with several other studies examining the neural basis of emotional versus non-emotional conflict. Processing of both kinds of conflict may rely on cognitive control-related brain areas [17], [49]. In particular, mechanisms underlying both emotional and non-emotional conflict detection have been localized to the dorsal ACC [15], [16], [50]. However, the present results are inconsistent with the older hypothesis that rostral and dorsal subdivisions of the ACC are devoted to processing emotional and cognitive conflict, respectively [45]. In particular, although we observed robust conflict-related activation in the dorsal ACC in both Emotion and Neutral conditions, no such patterns were observed in the rostral ACC, even when focusing exclusively on Emotion conflict.

The absence of emotion-specific conflict regions in the ACC during task processing may be surprising from the perspective of classic theoretical distinctions, but is actually relatively consistent with the prior literature. As discussed previously, original variants of the emotion Stroop actually target emotional distraction or even non-affective variables, and as such may not be appropriate for the study of emotional conflict, as suggested by recent conceptual analyses [14]. More recent studies that utilize conflict-based variants of the emotional Stroop and related tasks have been equivocal as to whether rostral ACC is either engaged, or associated with the detection (rather than resolution) of emotional conflict [15], [16]. Additionally, in one recent study rostral ACC activity during the emotion-conflict Stroop was dependent on the trait anxiety level of participants [23]. Thus, the current study adds to prior literature in suggesting that caution is warranted regarding whether the rostral ACC should in fact be associated with emotion conflict processing per se. Instead, further investigation of this region is needed, that focus on examining potential alternative accounts such as emotional distraction, conflict resolution, and individual trait anxiety.

In contrast to the pattern in the rostral ACC, there were significant effects of emotion on activation in a number of regions associated with cognitive control functions, including the dorsal ACC and lateral PFC. Interestingly, these effects were observed as significant condition (Emotion > Neutral) and conflict (Conflict > No Conflict) effects, without a significant condition × conflict interaction. In other words, the emotion effects were additive to conflict, rather than interactive, which suggests two independent mechanisms. At first glance, this pattern seems somewhat counter-intuitive, since the presence of affectively-valenced content did not selectively modulate the magnitude of the conflict effect, but instead increased activation equivalently on both high and low-conflict trials. Nevertheless, the pattern may actually be fairly consistent with interpretations regarding the nature of emotional conflict and control.

In particular, a key feature of the Emotional AX-CPT is that in the Emotion condition, there should be a relatively automatic, but task-irrelevant, subjective emotional reaction to the affective content present in the probe. This emotional reaction is task-irrelevant because correct response selection requires consideration only of the cognitive classification of the probe as having positive or negative content (and in integrating this information with cue classification). Indeed, the subjective emotional response to the probe, which may automatically trigger a tendency to activate the associated facial expression, can lead to an additional source of response uncertainty. For example, if viewing a negatively valenced probe stimulus triggers a tendency to make a frown expression (or likewise, if viewing a positively valenced probe stimulus triggers a tendency to smile), confusion can be generated regarding whether this “expression tendency” is appropriate for the current trial (i.e., correct on AX but incorrect on BX trials). Under such circumstances, from a cognitive control perspective, the optimal task strategy would be to suppress any subjective emotional responses that might be experienced in order to reduce response uncertainty. Because such task-irrelevant emotional response tendencies can occur on all trials in the Emotion condition, there would be generally higher cognitive control demands in this condition relative to Neutral.

In addition to the additive effects of emotion and conflict observed in regions associated with cognitive control, this same pattern was also present in the left amygdala, at least under an adjusted statistical threshold. The amygdala has typically been thought of as an emotion processing region whose activity, in conflict, distraction, and regulation paradigms, will reflect the emotional valence of stimuli, rather than tracking cognitive control demands [16], [51], [52]. However, prior findings of amygdala activity associated with increased cognitive control have also been repeatedly observed, although they typically receive less attention in the literature. For example, in one study increased amygdala activation was associated with improved behavioral performance during working memory, selectively under high-load conditions [53]. This finding, and others [54], [55], [56], supports alternative theoretical views of amygdala function, in which this regions is postulated to mediate general vigilance/goal-relevance-detection processes that contribute to enhanced cognitive performance as well as processing of emotional demands [57], [58]. The pattern of left amygdala activation in the present task – associated with both emotion and conflict-processing – might be better characterized by such an explanation, especially considering that emotional information must be evaluated for valence, while at the same time suppressing subjective emotional responses, in order to optimally perform the task.

Beyond the main effects of condition and conflict, a number of regions also exhibited distinct patterns of activity as a function of the type of interference present. As in the original AX-CPT, the emotional AX-CPT involves non-target trials eliciting conflict via two different forms of interference: AY trials, where interference is cue-based and relatively top-down in nature, and BX trials, where interference is probe-based and relatively bottom-up in nature. A number of frontal and parietal regions associated with cognitive control demonstrated significant trial effects in the present study, primarily because of BX > AY activity early in the trial (with comparable activity levels late in the trial). Previous studies of the AX-CPT have observed similar patterns of activation within the lateral PFC and other regions, demonstrating the robustness of the effect [46], [47]. The pattern of activity is typically interpreted as reflecting the higher degree of interference expectancy associated with B-cues (i.e., associated with non-target responses) relative to A-cues (i.e., associated with target responses), and thus increased demands for proactive cognitive control [28]. The current study extends this finding by demonstrating that this interference expectancy effects can be exhibited during emotional as well as non-emotional AX-CPT conditions. As such, the current results support the general notion that participants utilize the same types of proactive control strategies even when experiencing high demands for such control as a result of emotional conflict.

The emotional AX-CPT paradigm presented in the present study, and the use of emotional facial expressions as a response modality more generally, have the potential to provide a more naturalistic technique from which to probe emotional conflict, relative to the previous laboratory paradigms that have been used. Facial expressions have direct, automatic associations with different emotional experiences [59]; thus, they potentially provide a performance measure that is a more sensitive index of both trial-by-trial fluctuations and individual differences in emotional processing. In the present study we were not able to obtain behavioural performance measures due to technical difficulties, but future studies capitalizing on this technique should explore this possibility (e.g., via simultaneous EMG and fMRI recordings). Additionally, using facial expressions as responses permits elicitation of conflict via S-R interference, which is a robust form of interference that has nevertheless been understudied (relative to S-S interference) in the domain of emotion. The utilization of facial expressions as a response modality provides a potential means to probe emotional conflict via S-R interference in other paradigms as well, such as the Stroop adaptations utilized by Egner and colleagues [16], [22]. For example, in Stroop conditions that require participants to make facial expressions to semantically associated words (e.g., “smile”, “frown”) while ignoring irrelevant but superimposed affectively-valenced pictures, it would be possible to manipulate congruency in an analogous manner to that examined here.

One of the advantages of developing adaptations of the Stroop and related paradigms (e.g., Flankers, Simon) that include facial expressions as a response modality is that it would permit exploration of experimental manipulations not easily implemented in the AX-CPT. In particular, conflict-related shifts in control state (e.g., conflict adaptation or resolution effects) have been profitably examined through manipulation and examination of trial-by-trial sequential effects [60], changes in relative trial frequencies [61], and other similar effects. As a means of eliciting emotional conflict in a naturalistic, ecologically valid manner, the S-R incompatibility elicited through facial expression-based responding has the potential to be exploited in a similar variety of experimental manipulations, contributing to our knowledge of the behavioural and neural mechanisms underlying emotional conflict processing. It is our hope that this technique may provide one direction by which investigations of emotional conflict may approach the rigor and sophistication of similar research within the more traditional realm of cognitive control.

## Supporting Information

Figure S1 (a) Error rates and (b) response onset times measured via EMG in the Emotion AX-CPT, from Chiew & Braver (2010), as a function of Condition (Emotion vs. Neutral) and Conflict (high vs. low).
(DOCX)

Table S1 Centres of mass for cognitive control network (CCN) regions of interest (ROIs) used to mask the neuroimaging data.
(DOCX)

Table S2 Coordinates for hand-drawn emotion/reward-related (EMO) regions of interest (ROIs) used to mask the neuroimaging data.
(DOCX)

Table S3 Activity in areas defined by task conflict (high > low conflict) within anatomically defined ROIs with discernable errors eliminated, and comparable areas with all trials included (from Table 1).
(DOCX)

Text S1 (DOCX)
