# Supplementary material for: Neural Circuitry of Emotional and Cognitive Conflict Revealed through Facial Expressions
Source: PLoS One. 2011 Mar 9;6(3):e17635. doi: 10.1371/journal.pone.0017635 (PMC3052361; doi:10.1371/journal.pone.0017635)
Supplement: Figure S1 — (a) Error rates and (b) response onset times measured via EMG in the Emotion AX-CPT, from Chiew & Braver (2010), as a function of Condition (Emotion vs. Neutral) and Conflict (high vs. low). (DOCX) [file pone.0017635.s001.docx]

Figure S1a.

Figure S1b.
