# Supplementary material for: Neural Circuitry of Emotional and Cognitive Conflict Revealed through Facial Expressions
Source: PLoS One. 2011 Mar 9;6(3):e17635. doi: 10.1371/journal.pone.0017635 (PMC3052361; doi:10.1371/journal.pone.0017635)
Supplement: Table S1 — Centres of mass for cognitive control network (CCN) regions of interest (ROIs) used to mask the neuroimaging data. (DOCX) [file pone.0017635.s002.docx]

| **Study** | **x** | **y** | **z** | **mm^3^** |
| --- | --- | --- | --- | --- |
| **Owen et al. (2005)** |  |  |  |  |
| Lateral premotor (BA 6) | 28 | 0 | 52 | 10-mm sphere |
|  | -26 | 2 | 52 | 10-mm sphere |
| Dorsal cingulate/SMA (BA 32, 6) | -2 | 12 | 42 | 10-mm sphere |
| DLPFC (BA 46, 9) | 42 | 32 | 30 | 10-mm sphere |
| Ventrolateral PFC (BA 44) | -50 | 12 | 8 | 10-mm sphere |
|  | -62 | 0 | 14 | 10-mm sphere |
| Frontal pole (BA 10) | -38 | 44 | 20 | 10-mm sphere |
|  | 36 | 46 | 18 | 10-mm sphere |
| Medial posterior parietal (BA 7) | 12 | -64 | 48 | 10-mm sphere |
| Inferior parietal lobe (BA 40) | 30 | -58 | 42 | 10-mm sphere |
|  | 38 | -46 | 38 | 10-mm sphere |
|  | -34 | -48 | 38 | 10-mm sphere |
| **Wager & Smith (2003)** |  |  |  |  |
| BA 10, 9, 46, 47 | -32 | 34 | 22 | 10-mm sphere |
| BA 9, 6 | -45 | 7 | 32 | 10-mm sphere |
| BA 40, 39, 7 | -37 | -51 | 41 | 10-mm sphere |
| BA 9, 10, 46 | 36 | 36 | 28 | 10-mm sphere |
| BA 7, 40 | 31 | -59 | 43 | 10-mm sphere |
| BA 47, 10, 11, 13 | 34 | 31 | -4 | 10-mm sphere |
| BA 7 | -12 | -70 | 46 | 10-mm sphere |
| BA 6, 32, 8 | 0 | 11 | 49 | 10-mm sphere |
| BA 6 | 27 | 0 | 56 | 10-mm sphere |
| BA 6, 9, 44 | 45 | 1 | 29 | 10-mm sphere |
| BA 6 | -28 | -4 | 56 | 10-mm sphere |
