# Supplementary material for: Neural Circuitry of Emotional and Cognitive Conflict Revealed through Facial Expressions
Source: PLoS One. 2011 Mar 9;6(3):e17635. doi: 10.1371/journal.pone.0017635 (PMC3052361; doi:10.1371/journal.pone.0017635)
Supplement: Table S2 — Coordinates for hand-drawn emotion/reward-related (EMO) regions of interest (ROIs) used to mask the neuroimaging data. (DOCX) [file pone.0017635.s003.docx]

| **Region** | **x** | **y** | **z** |
| --- | --- | --- | --- |
| Insula | 33 to 41 | -22 to 23 | -8 to 20 |
| Caudate | 2 to 17 | -2 to 23 | -7 to 25 |
| Putamen | 12 to 30 | 20 to -21 | -8 to 16 |
| Amygdala | 15 to 30 | -10 to 1 | -22 to -11 |
| Nucleus accumbens | 3 to 13 | 4 to 15 | -3 to -14 |
| Substantia nigra | -12 to -6, 5 to 16 | -11 to -24 | -6 to -15 |
| Ventral striatum | -15 to 9 | -9 to 8 | -11 to -3 |
| Paracingulate cortex | -5 to 7 | 36 to 45 | 5 to 15 |
