# Supplementary material for: Neural Circuitry of Emotional and Cognitive Conflict Revealed through Facial Expressions
Source: PLoS One. 2011 Mar 9;6(3):e17635. doi: 10.1371/journal.pone.0017635 (PMC3052361; doi:10.1371/journal.pone.0017635)
Supplement: Table S3 — Activity in areas defined by task conflict (high > low conflict) within anatomically defined ROIs with discernable errors eliminated, and comparable areas with all trials included (from Table 1). (DOCX) [file pone.0017635.s004.docx]

| **Coordinates** | **Cluster Size (mm^3^)** | **ROI** | **Z** | **BA** | **Area** |
| --- | --- | --- | --- | --- | --- |
| 0, 12, 49  (vs. all trials, 0, 11, 48) | 6399 | CCN | 3.43 | 32 | Dorsal ACC |
| 41, 29, 37  (vs. all trials, 41, 28, 35) | 1026 | CCN | 3.55 | 9 | R DLPFC |
| -44, 8, 34  (vs. all trials, -44, 8, 33) | 4401 | CCN | 3.62 | 9 | L IFJ |
| 46, 5, 32  (vs. all trials, 45, 5, 32) | 2565 | CCN | 2.86 | 9 | R IFJ |
| -50, 12, 3  (vs. all trials, -49, 13, 3) | 2052 | CCN | 3.47 | 47 | L IFG |
| 28, 0, 54  (vs. all trials, 28, 0, 54) | 6102 | CCN | 3.31 | 8 | R superior frontal (FEF) |
| -28, -1, 55  (vs. all trials, -28, -1, 55) | 6642 | CCN | 3.43 | 8 | L superior frontal (FEF) |
| 21, -60, 42  (vs. all trials, 18, -60, 43) | 19251 | CCN | 4.90 | 7 | R precuneus |
| -38, -53, 41  (vs. all trials, -37, -52, 40) | 5400 | CCN | 4.21 | 40 | L IPL |
| 11, -12, 6  (vs. all trials, 10, -12, 4) | 2889 | CCN | 3.80 | ---- | R thalamus |
| -10, -12, 7  (vs. all trials, -9, -11, 6) | 1053 | CCN | 2.06 | ---- | L thalamus |
| -31, -67, -44  (vs. all trials, -31, -67, -45) | 1026 | CCN | 2.79 | ---- | L cerebellum |
| 32, -60, -44  (vs. all trials, 32, -60, -44) | 1215 | CCN | 2.30 | ---- | R cerebellum |
| -34, 17, 0  (vs. all trials, -36, 17, 0) | 2646 | EMO | 5.08 | 47/13 | L anterior insula |
| 39, 20, 0  (vs. all trials, 39, 20, 0) | 2673 | EMO | 4.45 | 47/13 | R anterior insula |
| 10, -4, 17 | 1080 | EMO | 3.95 | ---- | R caudate |
