# Supplementary material for: Neural Circuitry of Emotional and Cognitive Conflict Revealed through Facial Expressions
Source: PLoS One. 2011 Mar 9;6(3):e17635. doi: 10.1371/journal.pone.0017635 (PMC3052361; doi:10.1371/journal.pone.0017635)
Supplement: Text S1 — (DOCX) [file pone.0017635.s005.docx]

Text S1.

*Global Error Rates*

Although poor video quality and technical difficulties prevented us from gathering enough video footage to use it as an evaluation of behavioural performance in the present study, we were able to visually inspect the data and obtain an estimate of global task performance from the footage that was intact: 88% accuracy, or 12% errors. Because this estimate is obtained with incomplete data, we did not break it down by trial type, but it is higher than error rates in our previous behavioural study ([1]; global error rate 5%). However, this global error rate is still low enough to indicate that participants generally stayed on-task. Moreover, our coding method was conservative, and did not differentiate partial errors or missed responses from true errors. Thus, it likely reflects an over-estimate of the true error rate in the study.

We analyzed the fMRI data with and without these discernable errors included. Data with all trials included were used for the primary analyses of the present paper, but with discernable errors excluded we observed very few changes in the results of the conflict contrast. These regions are shown in Table S3 along with their respective conflict-sensitive region identified with all trials included. In the CCN mask, one region in the right cerebellum, 33 -62 -26, was identified with all trials included but did not reach significance with discernable errors dropped. In the EMO mask, areas in the bilateral DA midbrain (8, -17, -10 and -6, -18, -10) and in the left putamen (-16, 5, -2) were identified in the conflict contrast with all trials included but did not reach significance with errors dropped. Additionally, an area in right caudate (10, -4, 17) was identified as sensitive to conflict in the analysis when errors were dropped but not when all trials were left intact.

*Task Performance (from Chiew & Braver, 2010)*

Although video footage in the present study was too poor a quality to extract measures of behavioural performance, a previous study from our laboratory examined performance in the emotional AX-CPT and a non-emotional matched task using EMG to index expression responses in the zygomatic and corrugator muscles (indexing smiling and frowning responses respectively; citation). Behavioural performance from that previous study (in terms of error rates and response onset times for high-conflict [AY, BX] versus low-conflict [AX, BY] trials) is shown in Figure S1. As in the present study, two versions of the task were used (AX trials were either ‘SMILE’/positive picture, to which participants had to smile, or ‘FROWN’/negative picture, to which participants had to frown); performance measures (again, error rates and response onset times) were taken from the correct channel for each task version (zygomatic muscle for smile, and corrugator muscle for frown) and combined for presentation here.

The Emotion condition elicited behavioural indices of conflict in terms of response onset and error rates (significant effects of trial-type in both error rates [*F*(3,96) = 3.512, *p* = .018] and response onsets [*F*(3,96) = 10.472, *p* < .001]). This was also observed in the Neutral condition (significant effects of trial-type in both error rates [*F*(3,96) = 3.817, *p* = .012] and response onsets [*F*(3,96) = 41.898, *p* < .001]. These conflict effects did not differentiate Emotion and Neutral conditions (trial*condition interactions did not reach significance). However, when muscle amplitude was examined in the first second after probe presentation, amplitude differences between bottom-up conflict (BX) and non-conflict (BY) trials were larger for Emotion than Neutral conditions. These findings suggest that EMG measures may be more sensitive than traditional behavioural responses, and that while the primary influence of the emotion condition was a main effect, there were also subtle indications of an increased conflict effect. This behavioural data provides support for our interpretation of the neuroimaging data in the present study: that the task elicits conflict in both the Emotion and Neutral conditions, but that interaction between conflict and condition remains uncertain.

It should be noted that our previous EMG study and the present neuroimaging study have some design differences between them: (1) In the EMG study, Emotion and Neutral conditions were conducted between-subjects while in the present study they were within-subjects; (2) a larger subject sample was used in the EMG study than in the neuroimaging study (N=68 vs. N=24); (3) the EMG study required that participants perform the task at both long and short cue-probe delays (which ultimately did not lead to significant changes in behavioural performance) while the neuroimaging study kept cue-probe delay at a constant 3250ms; (4) the neuroimaging study involved alternating blocks of task and rest and a jittered ITI so that sustained and transient brain activation could be separated, while the EMG study involved continuous task performance with a constant ITI; (5) the Neutral tasks differed slightly in the probes used (in the EMG study, vowels and consonants were used as targets and non-targets, with digits as no-go probes; in the neuroimaging study, letters and numbers were used as the targets and non-targets, with a pre-specified punctuation mark as a no-go probe). However, we do not anticipate that these differences led to significant differences in the behavioural and brain activity elicited by the task.

Supporting Information: References

1. Chiew KS, Braver TS (2010) Exploring emotional and cognitive conflict using speeded voluntary facial expressions. Emotion 10: 842-854.

Supporting Information: Figure Captions

S1. **Emotion AX-CPT behaviour measured using EMG.** (A) Error rates and (B) response onset times measured using EMG, from Chiew & Braver (2010), as a function of Condition (Emotion vs. Neutral) and Conflict (high vs. low).
